# London’s Ultra Low Emission Zone and active travel to school: a qualitative study exploring the experiences of children, families and teachers

**DOI:** 10.1136/bmjopen-2024-091929

**Published:** 2025-03-03

**Authors:** Olivia Alliott, Esther van Sluijs, Rosamund Dove, Harpal Kalsi, Jessica Mitchell, Ian Mudway, Gurch Randhawa, Luke Sartori, James Scales, Helen E Wood, Chris Griffiths, Cornelia Guell, Jenna Panter

**Affiliations:** 1 MRC Epidemiology Unit, University of Cambridge, Cambridge, UK; 2 Wolfson Institute of Population Health, Queen Mary University of London, London, UK; 3 Asthma UK Centre for Applied Research, Edinburgh, UK; 4 Institute for Health and Primary Care, Queen Mary University of London Wolfson Institute of Population Health, London, UK; 5 Queen Mary University of London, London, UK; 6 MRC Centre for Environment and Health, School of Public Health, Faculty of Medicine, Imperial College London, London, UK; 7 NIHR Health Protection Research Units in Environmental Exposures and Health, and Chemical and Radiation Threats and Hazards, Imperial College London, London, UK; 8 Institute for Health Research, University of Bedfordshire Faculty of Health and Social Sciences, Luton, UK; 9 European Centre for Environment and Human Health, University of Exeter Medical School, Exeter, UK; 10 Wellcome Centre for Cultures and Environments of Health, University of Exeter, Exeter, UK

**Keywords:** Health, QUALITATIVE RESEARCH, Health policy, Health Equity, Lung Diseases

## Abstract

**Abstract:**

**Objective:**

Taking a qualitative approach, we aimed to understand how London’s Ultra Low Emission Zone (ULEZ) might work to change behaviour and improve health in the context of the school journey.

**Design:**

Primary qualitative study embedded within an existing natural experimental study.

**Setting:**

A population-level health intervention implemented across London.

**Participants:**

Purposive sampling was used to recruit children (aged 10–11 years) from ethnically and socioeconomically diverse backgrounds within an existing cohort study, Children’s Health in London and Luton.

**Methods:**

In-person and online interviews were conducted with 21 families and seven teachers from the children’s schools between November 2022 and March 2023. Verbatim transcripts were analysed drawing on Braun and Clarke’s reflexive thematic analysis and guided by realist evaluation principles to identify contexts, mechanisms and outcomes using NVivo.

**Results:**

Common context, mechanism, outcome (CMO) configurations were identified reflecting congruent narratives across children, parents and teachers, for example, current active travellers (context) reported reductions in pollution (mechanism) leading to improvements in health, including alleviated symptoms of asthma (outcome). These CMOs were broadly captured by two themes: (i) how you travelled before the ULEZ matters: the impact of travel mode on experiences of the ULEZ and (ii) your context matters: the role of socioeconomic position in experiences of the ULEZ. Participants highlighted the potential for the ULEZ to positively impact their choice of travel mode to school, experiences of the journey and their health. However, the impact of the ULEZ differed inequitably by journey length, travel mode before implementation and access to reliable and affordable public transport.

**Conclusions:**

The capacity for the ULEZ to both narrow and exacerbate inequities across different travel contexts suggests when developing such schemes, more emphasis needs to be placed on providing accessible and affordable alternatives to driving.

**STRENGTHS AND LIMITATIONS OF THIS STUDY**

- We conducted semistructured interviews with teachers, parents and children to gain a variety of perspectives.
- Using semistructured interviews with vignettes allowed participants to discuss the topic in their own terms.
- As our findings are context-specific, the implications for other regions or cities implementing similar schemes may vary based on local socioeconomic and infrastructural conditions.

## Background

 To address the simultaneous challenges of poor air quality, rising levels of non-communicable disease and climate change, policymakers are implementing interventions at various levels.^1^ Active commuting has been widely encouraged, with increased funding for walking and cycling infrastructure and policies such as Clean Air Zones (CAZs), where restrictions are placed on highly polluting vehicles.^2^ Few studies exist on the impacts of these measures on children,^3^ who are particularly vulnerable to air pollution due to their developing respiratory systems.^4 5^

Review-level evidence on the effectiveness of interventions to promote active travel to school reveals a lack of evaluation of policy interventions at the population level.^6^,^8^ This gap persists despite increased implementation and evidence demonstrating the potential effectiveness and positive equity impact of population-based approaches.^9 10^ In their review, Jones *et al* (2019) identified the role of context in determining the effectiveness of environmental and policy interventions as a significant area of scientific uncertainty in promoting school-based active travel.^6^ In response, it has been suggested that more focus should be placed on understanding the intervention mechanisms (what an intervention did and how people responded)^11^ and whether this varies by context (the physical, social, political or organisational setting in which an intervention was evaluated or in which it was implemented).^11 12^

The limited evidence has reported mixed or inconclusive results on the effectiveness of school-based active travel promotion. This is largely based on aggregate behaviour change and quantitative outcome measures.^13^ However, it is likely the impacts of these interventions vary by context, individual experiences of the interventions and the salience of the intervention among different groups.^14 15^ Qualitative methods are beneficial in understanding these,^16^ with guidance recommending that both quantitative and qualitative approaches are needed to explore the potential effects of interventions and routes to behaviour change.^17 18^

Responding to the gaps in existing evidence, we conducted a qualitative study embedded within an existing natural experimental study, Children’s Health in London and Luton (CHILL). A previous quantitative analysis examined the impact of the Ultra Low Emission Zone (ULEZ) on active travel to school, finding that children living in the intervention area were more likely to switch to active travel compared with those children in the comparator area.^19^ Taking a theory-based perspective, we use a realist lens to understand how the ULEZ might work to change children’s travel behaviour and improve health in the context of the school journey and wider policy system. We focused particularly on those who are most vulnerable to the effects of poor air quality, such as those from low socioeconomic backgrounds.

## Methods

This study was reported following the Standards for Reporting in Qualitative Research (SRQR) (see online supplemental file 1).^20^ Ethical approval for this qualitative study was granted by the main Queen Mary’s University London (QMUL) Ethics of Research Committee (QMERC2018/08).

### Children’s Health in London and Luton (CHILL) study

The CHILL study is a two-arm prospective parallel cohort study aiming to evaluate the impact of London’s ULEZ on air pollution and children’s respiratory health.^21^ The primary outcome is lung function growth and secondary outcomes include physical activity, cognitive development, mental health and quality of life. The study compares children attending primary schools within ULEZ or within catchment areas of ULEZ, with children attending primary school in Luton/Dunstable, an area with similar levels of pollution at baseline. At the start of the study, all participants were aged 6–9 years old; baseline health assessments were completed before the implementation of the ULEZ in April 2019. Follow-up assessments were conducted annually over the following 4 years.

A total of 3414 participants from 84 schools were recruited to the study, of which 1664 were based in London (from 44 schools).

### Implementation of the ULEZ

**Figure 1:**
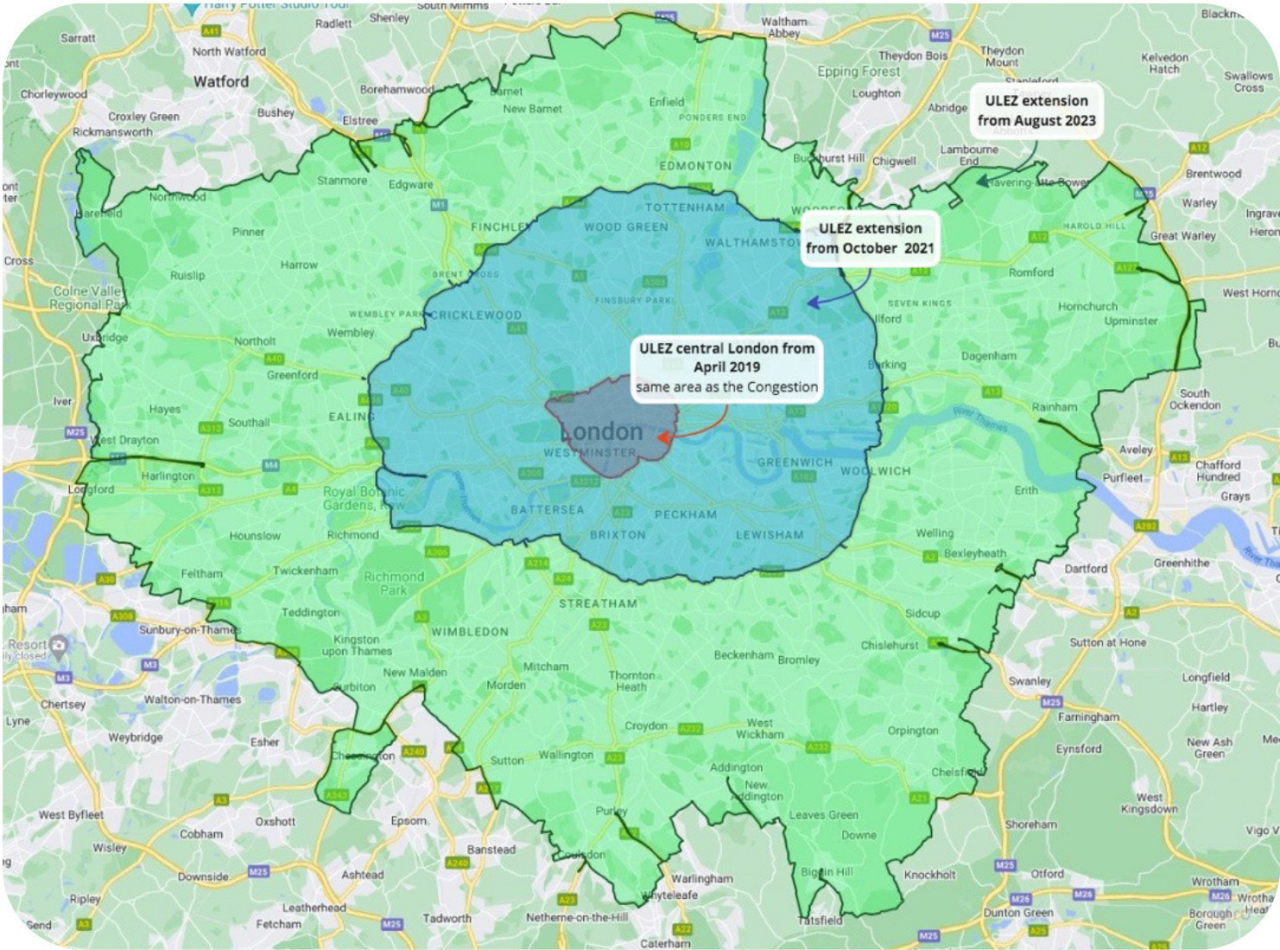
ULEZ boundaries 2019, 2021 and 2023. ULEZ, Ultra Low Emission Zone.

A CAZ is an area where access by some polluting vehicles is restricted or deterred, with the aim of improving air quality.^22^ Implemented in 2019, London’s ULEZ is a CAZ which initially covered central London, across the same areas as the then existing Congestion Charge (a £15 daily charge if you drive within the Congestion Charge Zone 7:00–18:00 Monday–Friday and 12:00–18:00 Saturday–Sunday).^23^ In October 2021, it was expanded to cover Inner London areas bounded by the North Circular and South Circular roads (figure 1) and was expanded again in August 2023 to cover almost all of Greater London.^24^ In this study, we refer to Central London as that within the boundaries of the Congestion Charge Zone.^23^

The ULEZ operates using automatic number-plate recognition to issue daily penalty charge notices to those entering the zone and not meeting set European emission standards.^25^ It applies to all vehicles 24 hours a day across the whole year, except for Christmas day. Money raised from the ULEZ is invested in the transport network and other measures to reduce pollution in London.^25^ There are specific exemptions in place, for example, for vehicles in the ‘disabled’ tax class. A scrappage scheme exists as part of the ULEZ, providing grant payments to successful applicants to scrap or retrofit vehicles that do not meet the emissions standards.^24^

### Participants and recruitment

Purposeful sampling was used to recruit participants from the existing London arm of the CHILL cohort. We asked school contacts to direct us to the most appropriate teacher in the school with knowledge of school-based travel behaviour and policies and interventions implemented in and around their schools. Baseline data were used to oversample children from ethnic minority backgrounds and those living in the context of socioeconomic deprivation. These groups have a higher exposure to poorer air quality and have been identified as especially vulnerable to the impact of poor air quality.^26 27^

Recruitment took place between November 2022 and February 2023. In the first instance, parents/guardians were contacted via telephone by a CHILL researcher they were familiar with. The aim of the call was to introduce the study and gauge parents’/guardians’ interest in participating. All those interested received an email with further information, including information sheets for both children and parent/guardians. Teachers and senior staff from the children’s schools were recruited via an initial phone call and subsequent emails. If no response was received, all participants were sent two follow-up emails and a final phone call before assuming they did not wish to take part.

### Data collection

Interviews were conducted with 21 families and seven teachers with a mix of face-to-face and online interviews in both groups. One researcher (OA) led the data collection, conducting participant interviews (between November 2022 and March 2023). Interviews were held at a time and place (home, school or online via Zoom) most convenient for the participant. Interviews with children took place with their parent/guardian in a dyad interview format (one child and at least one parent/guardian).

Each interview was recorded using a digital voice recorder. Prior to starting the recording, the interviewer took the time to ease participants into the interview process, recapping the information provided via email and answering any questions. Families signed a joint consent form including assent for children and consent for a parent/guardian. Those participating online provided e-consent. Where this was not possible, participants were sent paper copies of the consent form with a stamped envelope to return.

Interviews were conducted using a semistructured topic guide (both child/parent and teacher guide in online supplemental file 2) to aid the exploration of diverse practices and experiences of the school journey and lasted between 30 and 45 min. Vignettes (see online supplemental file 2) explored two contrasting hypothetical journeys to school (walking vs car use). These were used to elicit participant’s response and reaction to observing another’s behaviour. They additionally encourage participants to consider what they might do or feel in a similar context and are typically used in social research methods, including in realist evaluations.^28^,^30^ Teacher interviews were similar in format and aimed to understand school-wide travel behaviour. The interview process and materials were piloted with one family (n=1 parent and 1 child) before formal data collection began. Families and teachers received a £20 voucher to thank them for their time. Children were given blank versions of the vignettes which they could take home and colour in.

### Analysis

All interviews were manually transcribed verbatim, anonymised and imported into Nvivo software (Version 12 Pro, QSR International, Victoria, Australia) for analysis. One researcher (OA) led the analysis and consulted with the research team throughout the process. We did not recontact participants to check our interpretations.

The researcher first immersed herself in the dataset, listening to the audio recordings, reading the interview transcripts and making familiarisation notes. Taking a theory-based perspective, a realist lens was used to understand how the ULEZ might work to change children’s travel behaviour and improve health in the context of the school journey and wider policy system. This involved the coding and development of context, mechanism, outcome (CMO) configurations for each interview. Patterns across configurations were discussed in relation to the overall narrative of the data and our aim of understanding the role of the ULEZ in travel behaviour and health with a focus on the school journey.

To develop a deeper understanding of the lived experiences behind these configurations, we conducted a reflexive thematic analysis applying Braun and Clarke’s six-phase process for data engagement, coding and theme development, as follows: (1) data familiarisation and writing familiarisation notes; (2) systematic data coding; (3) generating initial themes from coded and collated data; (4) developing and reviewing themes; (5) refining, defining and naming themes; and (6) writing the report.^31^ As the researcher was already immersed in the data, initial codes were generated exploring surface (semantic) and underlying (latent) meaning in participants’ voices. Recognising that reflexive thematic analysis cannot be conducted in a theory vacuum, coding was both inductive and deductive, foregrounding participants’ perspectives and experiences while applying a realist lens.^32^ Initial codes were sorted into overarching categories, capturing multiple observations in the data, including non-observable entities and processes such as culture, socioeconomic circumstance and transport systems which may have influenced the impact of the ULEZ.

Candidate themes were developed and reviewed by rereading the collected extracts for each theme. Once satisfied, these adequately captured the coded data, and they were further refined, developing clear names and definitions for each theme. After a fully worked-out set of themes had been developed, the research team worked to produce a story about the data, reflecting the views and narrative of all participants. This is presented in the following sections.

### Researcher characteristics and reflexivity

To maintain reflexivity, the lead researcher kept a journal documenting a self-critical account of the research process, including interactions with participants and informal field notes of school visits. The analysis was guided by a team of researchers with expertise in health research focusing on children, travel behaviour and in-depth qualitative research. More detail on researcher positionality and methods to enhance rigour and trustworthiness are outlined in online supplemental file 3.

### Patient and public Involvement

Patient and public involvement (PPI) formed an integral part of the CHILL study, incorporating both formal and informal contributions. The main CHILL study design was developed through consultations with parents, headteachers, children from the study areas, and community groups such as ‘Mums for Lungs.’ A dedicated PPI group, composed of interested public members from previous research projects, was formed to ensure that the perspectives and well-being of participant children, caregivers and schools were prioritised throughout the study. This group provided feedback on study materials, supported recruitment and retention efforts, and offered advice on the dissemination of progress and findings. Additionally, the group included representatives who were members of the study’s Project Management Group (PMG) and Independent Study Steering Committee (ISC).

As part of a community outreach strategy, the study team engaged children from participating schools in interactive sessions on air pollution and health. The Centre of the Cell at Queen Mary University of London’s (QMUL) Science Education Centre delivered these sessions. Each year, new sessions were planned to explore different aspects of air pollution and health in alignment with the study’s objectives. This included the development of a floor-based board game, designed to encourage young people to discuss active travel and their school journeys (see online supplemental file 4).

## Results

**Figure 2:**
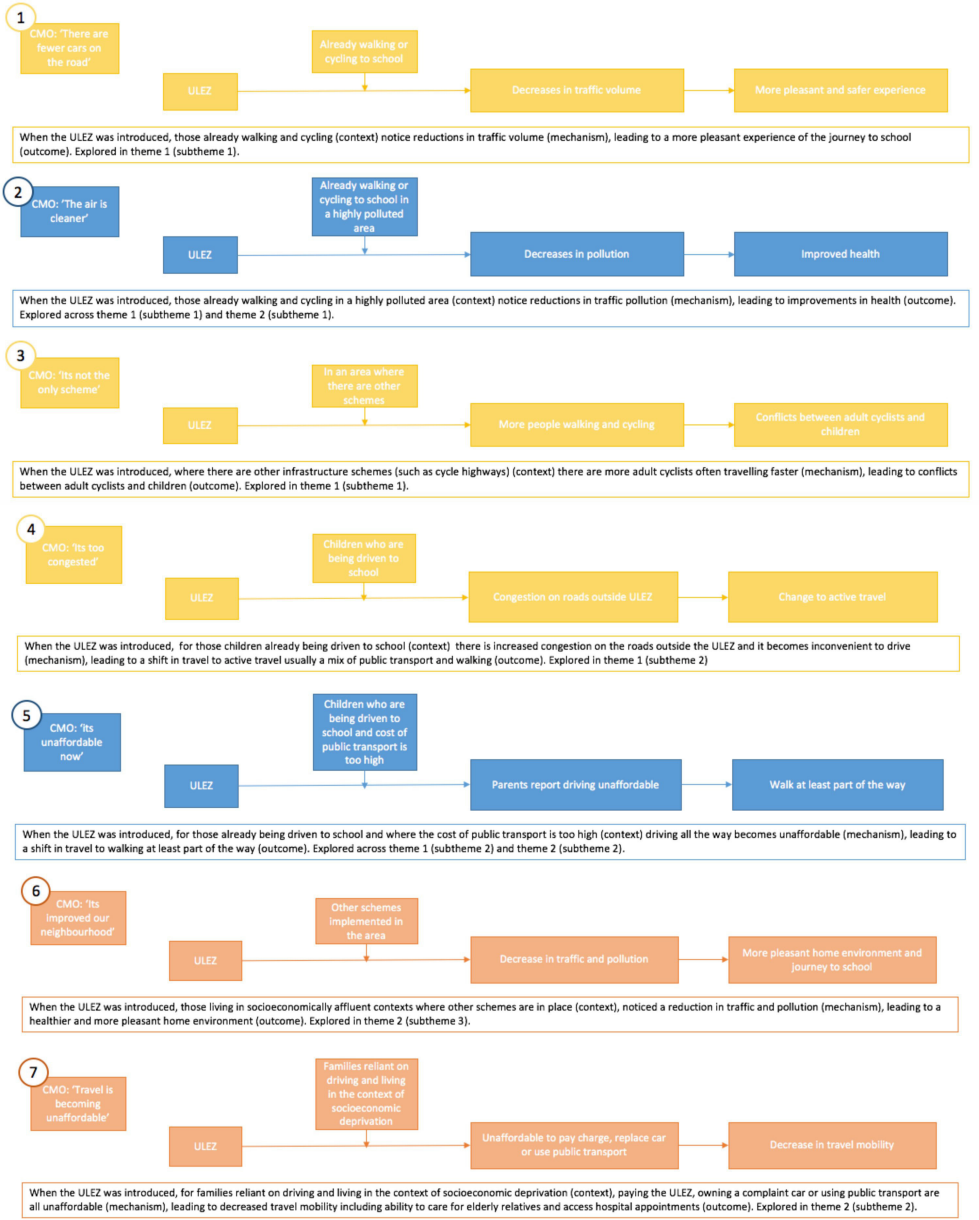
Common context, mechanism, outcome configurations which speak to how the ULEZ impacted travel behaviour and the school journey. Colour coding signifies which themes each configuration is explored across as follows: (1) yellow, theme 1; (2) blue, themes 1 and 2; (3) orange, theme 2. CMO, common context, mechanism, outcome; ULEZ, Ultra Low Emission Zone.

Common CMO configurations were identified which speak to how the ULEZ impacted children’s travel behaviour and journey to school. These configurations reflect narratives across two overarching, but intertwined, themes which were developed during the data analysis: (i) how you travelled before the ULEZ impacts the experience of the ULEZ, and (ii) your socioeconomic position impacts the experience of the ULEZ. No guardians were included in this study, from here onwards we refer to parents rather than parents/guardians. A visual summary of CMO configuration spanning both themes is presented in figure 2 and explored in more detail under each theme.

### Theme 1: how you travelled before the ULEZ impacts the experience of the ULEZ

This theme expands on CMO configurations 1–5, using travel mode to school before the introduction of the ULEZ as a context for differing responses to its implementation. We contrast active travellers and drivers, exploring differing experiences of the school journey.

#### Active travellers: experiences of the environment, safety and conflict between travel modes

Those who travelled actively before the implementation of the ULEZ tended to live more centrally, have a shorter commute and described having access to a dense active travel and public transport network in their area. For these participants, the ULEZ primarily improved their experience of the journey to school (configurations 1 and 2).

When discussing the impact of the ULEZ on walking, narratives focused on decreased levels of traffic volume and pollution as key mechanisms in making the journey more pleasant. One mother, who covered her face for religious reasons, described, “Yeah, but now it is nice to walk and so I cover my face, but even my skin cleared up and I feel better, and not got the smells and the pollution” (Parent 16). Many participants reported wanting to spend more time outside in response to the cleaner air, taking longer routes home and diverting via outdoor spaces such as parks. This allowed them to increase their active travel time and provided opportunities to engage in unstructured physical activity. One mother described,

So now what I do is, I like to take the longer route, which takes, like proper long, it takes about 15 min. And now sometimes I’ll also take my son to the park where there’s other mums there as well. So, you know, the kids get to run around for a little bit and play. (Parent 18)

Families who chose to cycle reported similar outcomes in relation to traffic and pollution, “I do remember you’d be close to a car clearly belching out smoke and, you know, I haven’t seen one like that for quite a long time so I guess it has done its job in taking those cars off the road and that has made things so much nicer” (Parent 7). Cyclists placed particular emphasis on investment in low emission buses (as part of the scheme). One parent described,

The buses obviously are low-emission and they’re much nicer to cycle behind. So like now you really notice when there’s a car that isn’t meeting the standard. (Parent 2)

A decrease in traffic on route and around the school fostered positive perceptions of safety and some parents were more likely to allow their child to travel independently, “the traffic was really bad and now it’s sort of lessened off a bit. I mean we let [M] make her own way home now which we wouldn’t have done in the past,” (Parent 15). Teachers also described students moving more freely around school.

Specific focus was placed on decreased levels of pollution as a mechanism impacting current and long-term health. This included improvements in breathing, “I say like biking in, obviously where there are less cars and less trucks on the street, it is great for your breathing” (Parent 7), in addition to alleviating symptoms of specific conditions such as asthma. For example,

For myself and people with asthma, because of the rule now there’s not as much fumes and smoke in the air, all these people with asthma can walk around the streets and not be coughing that much (Child 11).

A common narrative across active travellers was thinking about the ‘bigger picture’ and that to some extent, ‘we all benefit’. One young person described, “It helps everyone, it’s safer for humans because there’s less traffic. Oh, less deaths from cars. So yes, it impacts cyclists and pedestrians in a positive way” (Child 21). Teachers and parents further described the benefits of reduced pollution to children’s lung development. For example, “Children obviously, you know, we want to have as little pollution as possible in the lungs of our children and obviously for them to be as safe as possible” (Teacher 5).

In addition to these positive impacts, participants discussed how London’s changing travel environment had created tension between travel modes (configuration 3). Particularly, increasing numbers of cyclists, mainly work commuters and delivery bikes led to increased conflict “I personally feel it is more dangerous on the main road just towards the school, it’s now a cycleway, it’s very busy with cyclists and cyclists are extremely fast” (Parent 4). This experience was reiterated by teachers,

The bicycle traffic has grown since the ULEZ and is quite a danger at times. There are more of the electric bikes going all over the place, which are a little bit of a menace when you’re walking, and they’re literally all over the place. I mean, the main danger now to children on the streets is bikes around here. (Teacher 5)

#### Drivers: unaffordability, inconvenience and the compounding effects of other schemes

Participants who drove prior to the implementation of the ULEZ all reported a shift in travel mode, either to active travel or a hybrid journey using a mix of driving and active travel. These hybrid mode users tended to live outside of Central London and had a longer commute to school (configurations 4 and 5).

For many, the ULEZ being viewed as unaffordable was a primary mechanism motivating a shift in travel behaviour “the school is inside the zone so we would have to pay the charge every day, it is just not affordable, that is why we stopped driving” (Parent 7). For those with the longest commutes, a complete shift in travel mode was not always viable. Instead, these families chose to drive most of the way, park/get dropped off outside the ULEZ and walk the remainder of their journey. For example,

We used to drive and now we don’t drive the whole way, now we get dropped off and walk. We live outside the ULEZ so it’s really expensive to drive in (Child 6).

An increase in congestion on the roads outside the ULEZ was discussed as a further mechanism resulting in changes in travel, especially an increase in journey length and diverted traffic. One young person described, “it (the ULEZ) can be a really big inconvenience because you have to like, in my area the queues are so big now, when I want to like go in the car I have to like loop round the building to like park near our house” (Child 5). This was reiterated at the school level, where teachers outlined the inconvenience of driving after the ULEZ “it is just too inconvenient and takes too long, we did use to have some drivers but I think since ULEZ they have given up” (Teacher 2).

Some families reported a positive experience switching from driving to active travel.

The traffic, the A30 just stops/start and you can be there for half an hour and it was just getting too frustrating and this (the Tube) is just more a pleasant drive, a pleasant journey because [A] and I get to talk and we enjoy being driven by someone else. It costs me more to and from on Tube because of the peak hours and compared to what petrol is, but again like it’s just a much more enjoyable journey. (Parent 1)

In addition to a more enjoyable journey, participants described positive health benefits, primarily in relation to breathing conditions, due to cleaner air and increased physical activity. One parent highlights this below in relation to her daughter’s asthma,

Well [A]’s asthmatic as well so I think that exercise, walking and then catching the Tube and then less fumes, I think that has a great impact, like it’s really helped her, so she’s stopped, we’ve stopped using the inhalers unless she’s got a bit of a cold or something that kind of gives her more symptoms, and aggravates her cough, but other than that no, so it’s actually really helped her health wise and stuff. (Parent 20)

Diverted traffic was further discussed in the context of the school journey, as a time when many commuters take the same or similar routes. When reflecting on the planned expansion of the ULEZ, one parent explained, “now because of the diverted traffic everyone is trying to take the same main road to school, you just end up sat in loads of traffic, with the expansion it is just going to make things worse” (Parent 7). Participants placed the ULEZ in the broader policy environment, acknowledging how the combination of travel and traffic restriction schemes across London, including low traffic neighbourhoods and cycle superhighways, had impacted the travel landscape. One parent reflected,

It’s actually quite interesting how the London schemes have affected your travel habits, because you’re kind of forced financially and practically to adapt your method of transport to make it more convenient. (Parent 5)

Drivers went on to discuss the impact of the ULEZ on their future behaviour, suggesting they would be more likely to replace or sell their car if it were to become uncompliant. One participant described, “if my car became uncompliant I would definitely change it because I don’t want to pay £12” (Parent 8). The ULEZ was further reported to impact the amount participants used their car for non-school journeys, or whether they owned a car at all, “So, yeah, so on a day-to-day basis we use it less, but it would certainly influence my more, bigger decisions about what car to have” (Parent 5).

### Theme 2: your socioeconomic position impacts the experience of the ULEZ

Participants’ individual context further impacted their response to the ULEZ. This theme focuses on participants’ socioeconomic context and travel priorities in relation to the school journey, building on the differing experiences by travel mode outlined above (configurations 1–5). As part of this theme, we further explore the impact of the ULEZ on children’s home environment and broader journeys (configurations 6–7).

#### Living in deprived areas in Central London: it’s improved our health

Active travellers from socioeconomically deprived areas reported living in the most polluted parts of Central London and experiencing the greatest impact of reduced pollution levels. These families placed particular emphasis on the benefits to their present and long-term health, highlighting a favourable equity effect in this context (configuration 2).

we’re from the deprived areas you know, people that are from deprived areas are living at least 10 years shorter than somebody who was, you know, from a wealthy area. So, if we can do anything about these kind of situations then why not? Because in the long run it’s (the ULEZ) beneficial to us, we’re going to be living longer, you know the future generation is also going to be living longer. (Parent 18)

This was especially important for participants with existing health conditions. One young person spoke to this in relation to his asthma, explaining how the cleaner air allowed him to walk without breathing difficulties, “it’s just much nicer, you can walk around now and the air doesn’t hurt your lungs” (Child 11). Prior to the implementation of the ULEZ, participants described the imposing presence pollution had on their day-to-day life. When asked if she had been impacted by the ULEZ, one mother explained,

There was a time where I used to think that I was literally going mad because I’d sit there, I’d go anywhere and I could smell fumes, it was like I could smell it, I could taste it, literally taste it. It was so bad, I was stressed with it, I was crying at times. (Parent 16)

#### Living in deprived areas in Greater London: it’s unaffordable and inequitable

Families in Greater London from socioeconomically deprived areas reported different experiences compared with those living centrally. They highlight the capacity for the ULEZ to simultaneously narrow and exacerbate socioeconomic inequities. With longer school journeys, they relied more on driving and continued to drive part of the way after ULEZ implementation (configuration 5).

Ability to pay the charge was a major focus, especially in the context of the cost-of-living crisis. One young person described, “everyone’s going through a hard time because of the cost-of-living crisis, and then every penny counts, for people like us driving is a little bit expensive” (Child 9). This was linked to the idea that London is becoming financially ‘unliveable’ for many of its residents. When discussing the planned expansion of the ULEZ, a mother explained,

It’s going to be good for all of us, but at the same time like, it’s contributing towards making London a little bit, while it’s healthy liveable, it’s unliveable financially. (Parent 19)

While the ULEZ targets driving, the rising cost of public transport made shifting travel mode challenging (configuration 7). For some families, this meant driving was still the cheapest option. One participant described, “Well, the working classes pay for it (the ULEZ). Sadiq (Mayor of London) is making a lot of money, but I think where he went wrong is putting up the prices for the Tubes and the buses, it just makes it so expensive to get to school” (Parent 17). Another parent explained that public transport was not financially practical.

Public transport is actually quite quicker, yeah…the boys would love to travel on the train but the reason we use our car is because it’s cheaper for us, five of us to travel by car every day than on the transport. (Parent 6)

This was further emphasised at the school level, as illustrated in the quote:

we’ve got quite a lot of refugees and groups of, groups of our community that are staying with us temporarily, so we do have a lot that suddenly move out of the area so then they just have to take the cheapest mode of travel, so then they’re public transport, or they drive in because they’ve moved into a different borough and even with the ULEZ it is still cheaper than public transport, but they still want to stay at the school, so we do have a community of people that do use cars. (Teacher 6)

In addition to the school journey, the increasing cost of travel was reported to impact families’ broader travel mobility (configuration 7). This included making it more difficult to access health appointments and family members. One mother speaks about how the ULEZ had impacted the regularity of visits from her family.

My brother has a car so two times, he had to pay for the fine (ULEZ charge) so I felt really bad for them as well. Every time one of my relatives, if they have a car, they can’t bring the car in to where we live because of the charges. So, I think it’s not fair on them, yeah and use cause now they can’t visit as much. (Parent 14)

In addition to the daily ULEZ charge, participants highlighted further inequities in the cost required to replace their existing vehicle with one which meets the emission standards. One parent described how they had to take out a loan in order to replace their car using the scrappage scheme.

Yeah, so the scrappage scheme was good, so we got a bit of money back, and that helped, but I mean we ended up having to spend more money than we actually had, which meant we borrowed to buy a new car, and we had to have a car, because as much as we do use, as much as everything local is kind of walking distance and what not, we do travel out a lot, so we need a car. (Parent 9)

#### Living in more affluent areas: it’s about the convenience and experience of travel

Families in socioeconomically advantaged areas tended to live in quieter suburban neighbourhoods outside Central London. They emphasised the complex policy system of the ULEZ, noting how various schemes together impacted travel patterns and improved neighbourhood walkability. Families described living on quieter streets where the synergistic implementation of other traffic-calming measures (eg, low traffic neighbourhoods) created a more pleasant home environment and journey to school (configuration 6).

It wasn’t just the ULEZ, it was the changing traffic on like the smaller residential streets which made the journey more pleasant, yeah and actually things like widening the pavement which makes it easier for the families, prams, scooters, etc. to make it safer. (Parent 20)

In this context, convenience was a primary mechanism changing travel behaviour (configuration 4), with families tending to shift from driving to active travel, “it’s just more convenient on the train, [H] and I can chat more and it is just nicer than trying to navigate all the restrictions” (Parent 20). One teacher described this at the school level, explaining how more financially buoyant families could afford to switch to active travel modes such as cycling.

I would say that a lot of it’s to do with the demographic of the school, it is becoming a lot more middle-class lower down and, you know, those cargo bikes are a very middle-class, so I think these families can afford to switch to cycling, they can afford a bike and have a home where they can store it. (Teacher 3)

London’s changing travel landscape was a common narrative, making it hard to gauge the exact impacts of the ULEZ due to other travel policies and the wider policy environment. When discussing a decrease in traffic and pollution, one father described, “…maybe more because of these pilot schemes that close off subsidiary roads, I am not sure if it is because of ULEZ” (Parent 8). The COVID-19 pandemic was discussed as a further “spanner in the works” in determining the effectiveness of the ULEZ.

I don’t know how much impact it’s (the ULEZ) had, you know, measurably on people’s health, but certainly like the physical environment of the streets it’s really very different. Of course, lots of things have happened since the ULEZ, so we had like the pandemic and then now we have a lot of, there’s a lot of traffic that’s different, we have a lot of very high-speed electric bike traffic, it makes it really hard to gauge. (Parent 20)

While pleased with the changes in their local area, participants were concerned that traffic had been diverted to already congested main roads in less socioeconomically affluent areas. Many participants displayed a strong social conscience and were concerned that the benefits they experienced were at the cost of others. One parent described,

So, from our perspective I think it’s helped and I think it’s great, we live in a fairly quiet area off the main road, but I’m just mindful that it’s just a sort of kicking the can down the road and it’s just pushing it out to other parts of London. (Parent 7)

## Discussion

The ULEZ, introduced into Central London in April 2019, provided the opportunity to explore the impact of a population-level intervention on children’s travel to school. Using a qualitative approach, we aimed to understand how the ULEZ might change travel behaviour and improve health in the context of the school journey and wider policy system. Two interconnected themes were developed, reflecting the views and experiences of children, parents and teachers, discussed below in the broader literature context.

### Findings in context

#### How you travelled before the ULEZ matters, the impact of travel mode on experiences of the ULEZ

Previous research shows the ULEZ caused a positive shift to active travel, especially for those living farther from school.^19^ We found those with longer journeys relied more on driving and had a propensity to change, explaining why a modal shift is more likely among this group. Our findings highlight decreased convenience and increased costs as key mechanisms driving behaviour change, reinforcing that ‘stick’ strategies (negatively motivating behaviour) are effective in discouraging driving.^3^ Active travellers reported decreased pollution, traffic and noise, positively impacted their health, safety perceptions and time outdoors. This highlights additional benefits of the ULEZ and affirms systematic review evidence that Clean Air Zones (CAZs) have the potential to improve long-term health and reduce car-related injuries.^33^

The introduction of low-emission buses positively impacted cycling experiences. This supports research showing financial mechanisms reduce driving, while improving access, safety and space promotes active travel (acting as a ‘carrot’).^3^ Participants noted that in London’s changing travel landscape, the increase in cyclists made them the main cause of accidents on school journeys. This highlights the need to consider safety and space in promoting active travel and explains the mixed evidence on the ULEZ’s impact on total traffic injuries.^33^

#### Your individual context matters, the role of socioeconomic position in experiences of the ULEZ

It is currently argued the most equitable CAZs are those with expansive parameters and high restrictions on polluting vehicles.^26^ Our findings add nuance, showing positive equity impacts on experiences of traffic-related air pollution while highlighting the economic burden on those unable to afford cleaner vehicles. We further illustrate the equity impact of CAZs could differ according to journey length, travel mode before implementation and access to reliable and affordable public transport.

Despite the health benefits and potential equitable impacts of CAZs on vulnerable groups like asthmatic children,^26^ research shows these measures can reduce life satisfaction.^34^ Our participants’ decreased access to family and health appointments highlights how reduced travel mobility can worsen socioeconomic inequities when not implemented alongside affordable and well-connected active travel infrastructure.

Xiao *et al* (2024) note that overlapping strategies in London make it hard to attribute changes specifically to the ULEZ rather than other policies.^19^ Our participants speak to this from an equity stance, with those reporting living in socioeconomically affluent contexts more commonly speaking to the success of the combination of these schemes in their local area. This aligns with broader health literature highlighting inequities in provision and uptake as points for consideration in the planning and delivery of public health interventions.^35^

### Strengths and limitations

Focusing on the experiences of children, families and teachers, this study adds in-depth, contextual understanding to existing evidence on the impacts of the ULEZ on school-based travel.^19^ Exploring these experiences has advanced our understanding of how the ULEZ can both narrow and exacerbate socioeconomic inequities, as well as the equitable implementation of CAZs globally. The semistructured interview format and use of vignettes allowed participants to discuss the impact of the ULEZ on their terms, generating nuanced insights and shared experiences.^30^

While informative to the development of CAZs, our findings may not fully capture variations in school journey experiences and transport infrastructure beyond this context. However, this is consistent with a qualitative approach.^36^ Interviews were conducted between November 2022 and March 2023, meaning our results do not include participants’ experiences of the ULEZ expansion to the majority of Greater London (August 2023). Moreover, it is important to acknowledge the possibility of social desirability bias, especially in discussing such a politically controversial topic, and all possible explanations might not have been captured.

### Implications for research and practice

The transportation sector is one of the largest contributors to urban air pollution and has the potential to significantly reduce health disparities between socioeconomic groups.^26^ The ULEZ’s impact on travel equity underscores the need for accessible, affordable alternatives to driving when designing such schemes. Affordable, convenient active travel infrastructure is needed to support equitable mode shifts for long-distance travellers. Implementing CAZs alongside supportive active travel infrastructure is needed, as evidence suggests combining positive (carrot strategies for example, public transport promotion) and negative strategies (stick strategies for example, car use limits) is more effective at the population level.^3^

Research by Xiao *et al* (2024), accompanied by the experiences of our participants, indicates that CAZs like London’s ULEZ play an important role in the school journey and encouraging active travel.^19^ Expanding existing measures or implementing similar strategies in cities across the UK could help the Government to achieve its 2025 walk to school target^37^ and the Mayor of London’s objective of having 60% of children walking to school by 2026.^38^ As cities worldwide plan to adopt similar schemes, the learnings for this study and the ongoing evaluation of their impact across social and travel contexts are vital. Prioritising equity in these assessments, including analysing the impact on diverted traffic and potential inequities by sociodemographic factors in bordering areas, is crucial.^26^ The expansion of the ULEZ in August 2023 is an example of just one opportunity where this could be explored.

## Conclusion

Our findings show the capacity for the ULEZ to encourage a shift to active travel and positively impact participants’ experiences of the school journey. Through an exploration of the wider social and policy context of the ULEZ, we highlight the need to implement such schemes alongside accessible and affordable alternatives to driving.

## Supplementary material

10.1136/bmjopen-2024-091929 online supplemental file 1

10.1136/bmjopen-2024-091929 online supplemental file 2

10.1136/bmjopen-2024-091929 online supplemental file 3

10.1136/bmjopen-2024-091929 online supplemental file 4

## Data Availability

Data are available upon reasonable request.
