# Supplementary material for: London’s Ultra Low Emission Zone and active travel to school: a qualitative study exploring the experiences of children, families and teachers
Source: BMJ Open. 2025 Mar 3;15(3):e091929. doi: 10.1136/bmjopen-2024-091929 (PMC12010349; doi:10.1136/bmjopen-2024-091929)

**Supplementary File 2:** Interview topic guides and vignettes

**Interview topic guide for parents and children
Version 1.2, November 2022**

**1. Initial interview**

**1.1. Introduction**Explain purpose of research project
Explain audio recording procedures
Ensure parent/child has copy of participant information sheet (have read and understood)
Answer any questions
Complete consent/assent form
Commence audio recording

**1.2 Warm up questions to ease into interview (assess if needed from introductory dialogue)**

For the child: can you tell me a little bit about yourself, how old are you? What do you like to do for fun? Tell me about your Christmas break?

For the parent: Ask parent to say a little about themselves. Ask how their day has been or about their work, is this a typical day etc.

**Main Interview Questions**

**1.3. Typical journey to and from school**

For parent: Could you describe your usual journey to and from school.

Prompts: Usual origin(s) for journey to school, any vias on the way or way home

For child: What modes of travel do you use to get to school? What way to do you usually go?

Is that always the same?

**1.4. Reasons for these choices and alternatives available**

For parent/child: Why do you choose this route and (combination of) mode(s)?

Could the journey be made by other routes or other (combinations of) modes?

What factors influence the choice between these options?

Prompts to be used if necessary: (child friendly adaptations as examples)

Availability of other modes

Comfort
Convenience (because it’s easy)
Cost
Distance (because its short/quick)

Environmental concerns (because we care about the environment)
Exercise

Habit
Need to carry bags, instruments, other children
Safety
Time
Trip-chaining
Weather

**1.5. Variations on the typical journey and reasons for those variations**

For parent/child: Could you describe any variations in your typical route/journey to and from school? What are the reasons for this?

prompts to be used if necessary:

When starting or finishing work at different times

When transporting children or other passengers, shopping, trip-chaining
Weather conditions

**1.6. Potential for change, barriers and facilitators**

For parent/child: Thinking back over the last few years, has anything about the journey changed?

Any expectation or intention of changing travel mode(s) in the future?
What factors act as barriers to making that change? Or to the journey?
What factors would facilitate that change?
Why do you think other commuters make other travel choices?

Prompts listed above under 4 to be used as necessary

**1.7** **Vignettes to explore travel experiences (child uses picture vignettes)**

1. Decrease car use

I mean it’s really in the last couple of years my attitude’s changed. There are lots of little residential streets we can use on the journey to school so they are not so busy with traffic. And I do that so that I can take my son to school and then carry on with my journey to work. The real change was when we noticed fewer cars around but it came gradually, it just makes the journey so much nicer when I’m walking and he’s scooting, not watching all the cars queuing right beside us or having all the fumes. Before there would be major queues at the junction up to the school. It’s still busy there but it seems less busy to me now at least. They’ve also widened the pavement and lowered it in a few places, that’s much easier if I’ve got the pram for my youngest too; you’ve got to eyes in the back of your head with him and then me trying to concentrate on the traffic too. I don’t think much has changed around school. I mean they have bike racks and places for helmets but for me it’s mostly the middle part of the journey which is still the worst, where there are more lorries and cars; that’s the part where I have to pay most attention.

2. Continuation of car use

The last few years I’ve been driving and I love driving, I drive everywhere. Having the car gives you much more freedom, especially with a child. One reason for driving is that I need the car for work and I drop her off at school and then carry on. Deciding between driving and other options is like a balance between the convenience of a car which can literally get you from door to door, with trying to do the green thing and using the bus or tube or cycling. Comparatively cycling is not actually much different to the car because I’d still leave the same time and I’d probably arrive at the same time because of that last bit coming up to the school the traffic is probably comparable. It’s just the slight inconvenience of cycling and having to change when you come to work with the bike or bring all the safety gear on top of everything else, bags and the sports kit.

**Accompanying question:**

I wonder if you could both describe your immediate reactions to these stories…?

Child: Can you describe what you see in both of these pictures?

Parent: What are your immediate reactions to these stories?

Prompts to be used if necessary for parents and children:

Vignette 1

Do you think person 1 enjoys their journey? Why?

Do you agree with person 1’s views on the journey?

Can you relate to the use of different transport modes in different weather conditions?

Are there any other reasons you might choose different transport options?

Prompts to be used if necessary parents and children:

Vignette 2

Why do you think person 2 enjoys using their car?

Why do you think person 2 feels that having a car gives them more freedom with children?

From your experience, do you think there are any other reasons for driving to school and work?

What do you think about person 2 talking about the inconvenience of needing a change of clothes when you cycle to work?

Are there any other inconveniences related to cycling that you have experienced?

How important is convenience to your own travel choices?

**1.8. Perceptions of the ULEZ (Depends if school location is on the border of the ULEZ or in the central area of the zone)**

Parent/child: I now have some questions about the ULEZ in London, is this something you have both heard of?

**If yes**…Ask parent and child to describe what they understand the ULEZ to be.

**If no**…the ULEZ is a charge for driving polluting vehicles (cars, vans, buses) in central London…

Child: How do you feel about charging cars who drive near your home and school?

Prompts: is it a good/bad idea? Can you think of any advantages/disadvantages?

Parent and child: Can you think of any changes to your behaviour?

****invite child to stay for the remaining questions or leave depending on their knowledge of the ULEZ and engagement with the topic****

Parent: Could you tell me about your impressions of the charge? And your experiences of the charge?

Parent (and child if they have stayed):

Has anyone you know paid the charge for any journeys?

What sort of journeys were they?

Have you noticed a change in your own journeys (if addressed above, any further changes)? Or the journeys of others?

Prompts:

Advantages/disadvantages of the charge (walking, cycling or bus use)

Factors would/ do prevent/encourage alternatives modes of travel to the car (walking, cycling or bus use)

Identification of groups that have particularly benefitted from the charge

**1.9. Close**
End audio recording
Thank for participating, ask if the parent or child have any questions or concerns

**Teacher Interview topic guide
Version 1.2, November 2022**

**1. Initial interview**

**1.1. Introduction**Explain purpose of research project, “today I will be asking questions about the environment outside your school, your school’s policies and procedures about the journey to school and if and how this has changed over the last few years”
Explain audio recording procedures
Ensure teacher has a copy of participant information sheet (has read and understood)
Answer any questions
Complete consent/assent form
Commence audio recording

**1.2 Icebreaker questions (assess if needed)**

Can you tell me a little bit about your school and your role at the school?

Chat about how the term is going and school plans in the lead up to Christmas/February half term/Easter holidays.

**Main body of the interview**

**1.3. School environment for travel to school**

How would you describe pick up and drop off times to me or someone else who didn’t know the school?

**1.4. School or local policies on travel**

What facilities exist at the school for parents/children to support different modes of travel?

Are they well used/overcrowded? Are they recent additions or long standing?

Do you think the local area is conducive to children walking or cycling to school?

Do you think local councils support children walking or cycling to school?

**1.5. Potential for change**

Thinking back over the last few years, has anything about the journey changed for parents/teachers/children?

**1.6. Roles and responsibilities**:

Do you think there is anything more which could be done to encourage children to walk, cycle or use alternatives to the car?

Prompt, by schools or local councils or the government (at what level do you think this should be addresses?)

Do you know of any expectation or intention of schools to help children change travel mode(s) in the future?
What factors might act as barriers to making that change?

Prompts:

Money, funding

Time

Local environmental constraints?

What factors might facilitate that change?

(Prompts as above)

**1.7. Perceptions of the ULEZ**

I now have some questions about the ULEZ, is this something you are familiar with? If no, explain.

Could you tell me about your impressions of the charge?

Has anyone you know paid the charge for any journeys?

What sort of journeys were they?

What do you think of the charges?

Prompts:

Advantages/disadvantages of the charge (walking, cycling or bus use)

Factors would/ do prevent/encourage alternatives modes of travel to the car (walking, cycling or bus use)

Identification of groups that have particularly benefitted from the charge

Relate back to earlier discussion around changes in travel behaviour

**1.8. Close**
End audio recording
Thank for participating, ask if they have any questions or concerns.

**Interview Vignettes**

**
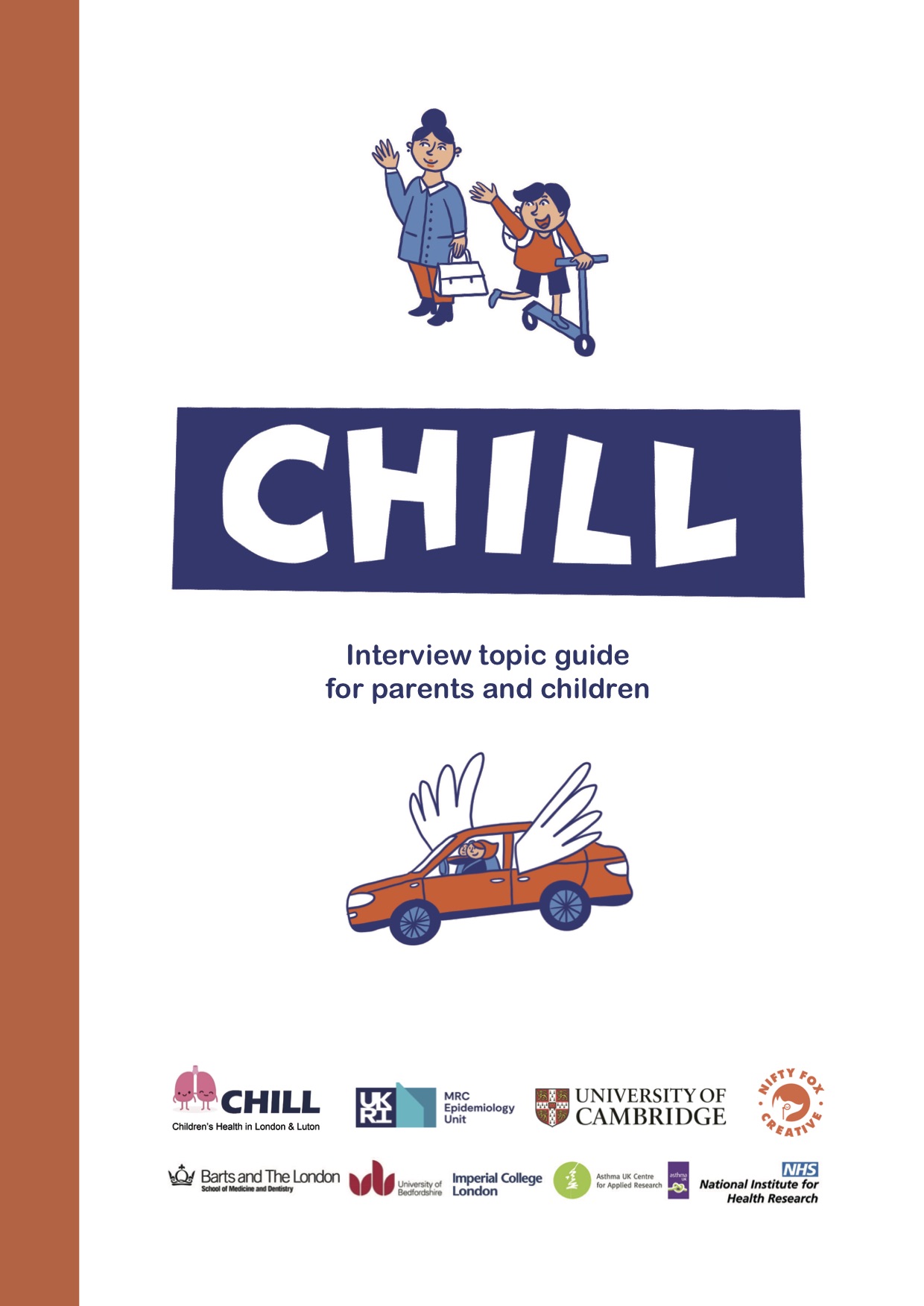
**


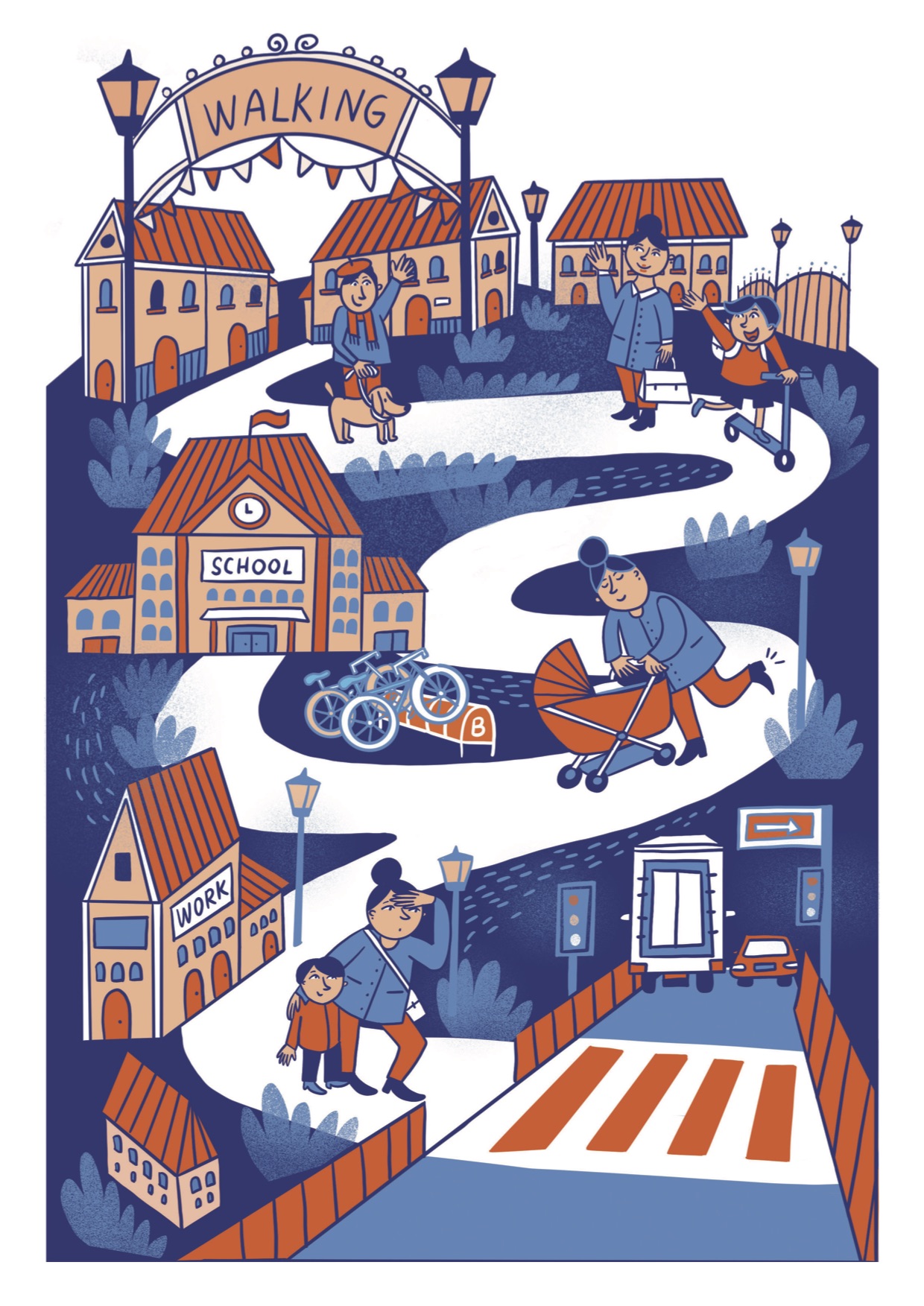


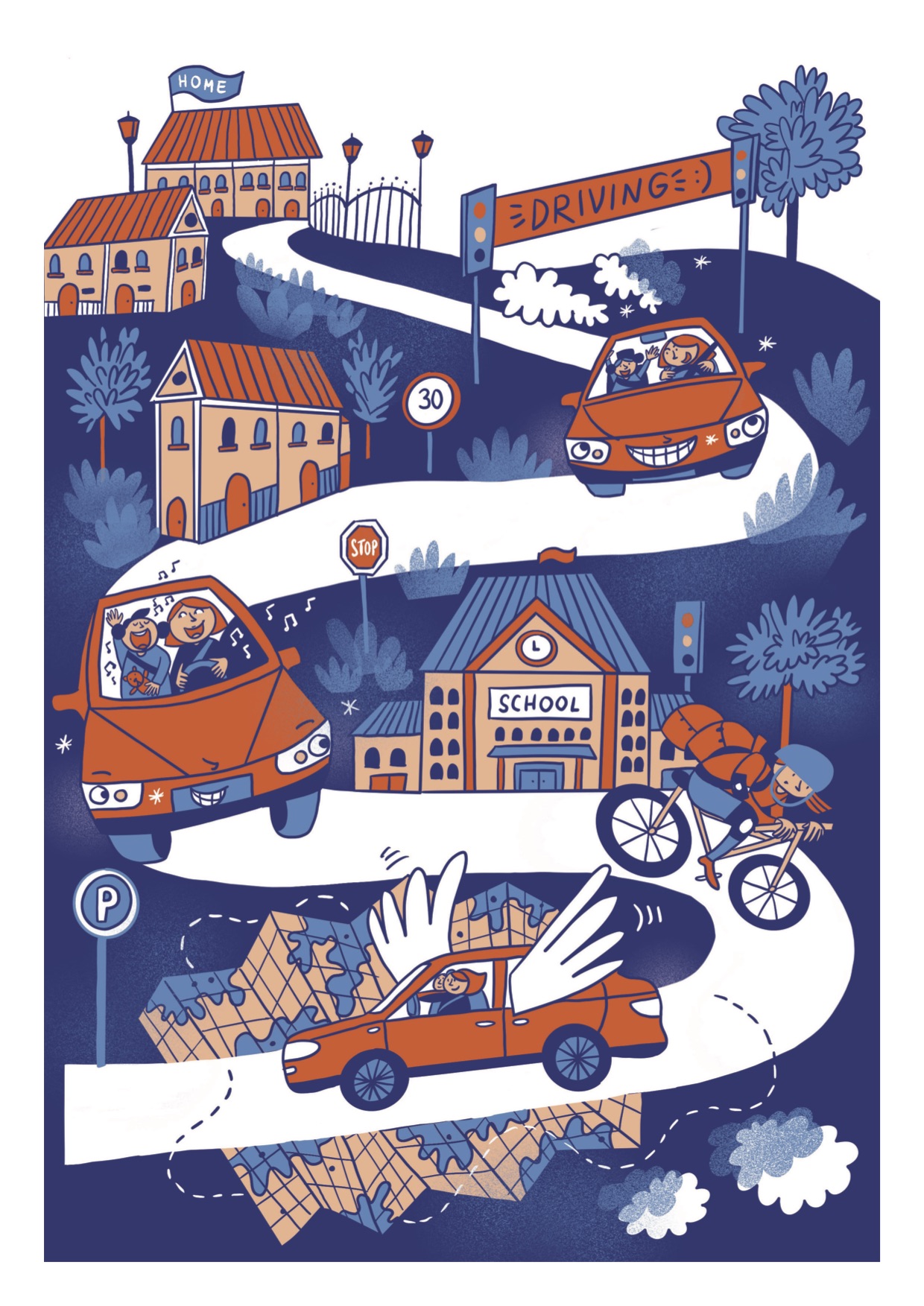


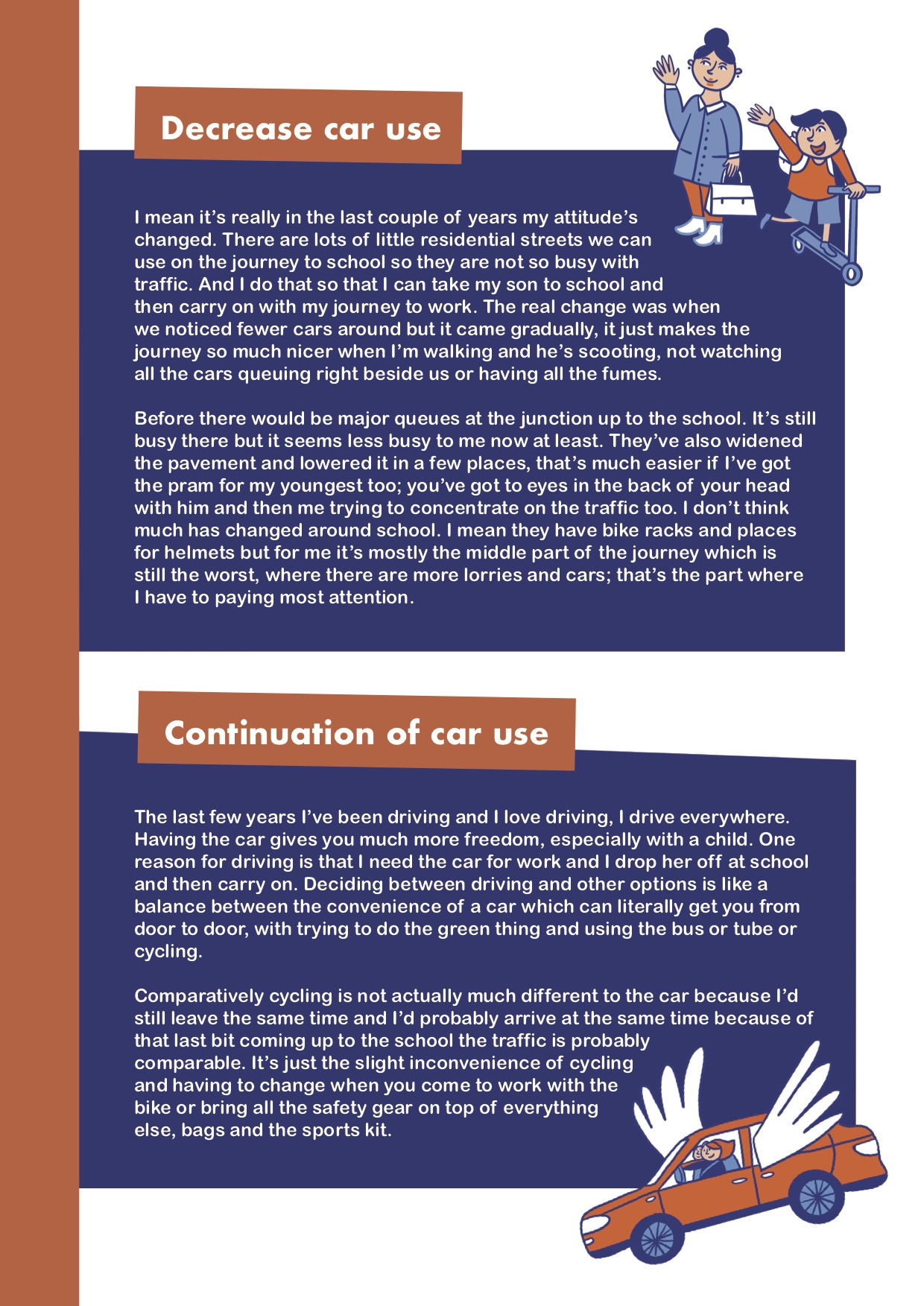

Supplement: online supplemental file 2 [file bmjopen-15-3-s002.docx]
