# Supplementary material for: London’s Ultra Low Emission Zone and active travel to school: a qualitative study exploring the experiences of children, families and teachers
Source: BMJ Open. 2025 Mar 3;15(3):e091929. doi: 10.1136/bmjopen-2024-091929 (PMC12010349; doi:10.1136/bmjopen-2024-091929)
Supplement: online supplemental file 3 [file bmjopen-15-3-s003.docx]

**Supplementary file 3:** Researcher positionality and methods to enhance credibility and trustworthiness

3.1 Researcher positionality and reflexivity

The researcher leading on the data collection and analysis was a white female of middle socioeconomic position (SEP) who was not previously known to participants as part of the wider CHILL study. Whilst she did not reside in London, she had experience of active travel, public transport and driving within central and greater London and took the opportunity to explore the environment around the schools and participant’s homes when conducting in-person interviews. The researcher acknowledges her interest in the role of SEP in physical activity and how this had the potential to sensitise the analysis. To encourage reflexivity and improve credibility, the analysis was guided by a team of researchers with expertise in health research focusing on children, travel behaviour and in-depth qualitative research (including with families) within large scale evaluations, and social theoretical approaches to behaviour change.

To maintain reflexivity, the lead researcher kept a journal documenting a self-critical account of the research process, including her interaction with participants and informal field notes about her experiences of visiting the schools, perceptions of the school environment and observed travel behaviours of students. Peer debriefing was used involving continual discussions about the research process and reflecting on researcher’s positionality. Braun and Clarke emphasise that quality reflexive thematic analysis is not about following procedures “correctly” but about reflective and thoughtful engagement with their data and the analytic process.^37^ In response, the research team aimed to conduct this analysis with theoretical knowingness and transparency, whilst being mindful of the philosophical sensibility and theoretical assumptions informing the analysis. To achieve this, and to increase rigour and trustworthiness throughout the analysis, the research team were guided by Lincoln and Guba’s (1985) trustworthiness criteria.^38^ The application of these criteria is detailed below.

3.2 The application of Lincoln and Guba’s (1985) trustworthiness criteria to the CHILL qualitative sub-study analysis.^1^

| **Criteria** | **Techniques and their application** |
| --- | --- |
| Credibility (internal validity) | Prolonged engagement with the transcripts   - Interviews were transcribed throughout data collection to allow for this - Transcripts were engaged with throughout the analysis   Triangulation   - Triangulation of researchers throughout the analysis - Triangulation of participant viewpoints, by collecting data from young people, their parents and teachers   Peer Debriefing   - The analysis was conducted as a research team (as detailed in manuscript) - Feedback from the research team provided on all written documents including but not limited to: the study protocol, interview schedule, analysis plans and resulting research paper for publication.   Referential Adequacy   - An iterative approach was taken to data analysis - Raw data, codes and themes have been stored to show their development   Negative case analysis   - Data which contradicted the explanations emerging from the data was considered and discussed |
| Transferability (external validity) | Trick description   - The research process has been described in detail using the Standards for Reporting Qualitative Research (SRQR). |
| Dependability (reliability) | Dependability audit   - Raw data, codes and themes have been stored to show their development - An audit was kept of the developing “story” of the data - The process of enquiry was continually re-examined, including but not limited to: how the data is collected, how the data was kept and the accuracy of the data in addressing the research questions |
| Confirmability (objectivity) | Confirmability audit   - The data collection and analysis process were transparently reported following the SRQR guidelines. - An audit trail was kept detailing each stage of the data analysis and of the research team’s discussions throughout this process. - The studies limitations have been acknowledged in the main manuscript - Data was appropriately managed, including but not limited to: participant information, interview recordings and verbatim transcripts of interviews |
| All four criteria | Reflexivity   - The researcher leading the data collection and analysis kept a reflexive journal documenting their positionality, notes of specific assumptions/subjectivities and an audit trial documenting decisions and choices made throughout the study. |

1. Lincoln Y, Guba E. *Naturalistic inquiry* Vol 1. Newberry Park, London, New Delhi: Sage Publications 1985.
