# Supplementary material for: London’s Ultra Low Emission Zone and active travel to school: a qualitative study exploring the experiences of children, families and teachers
Source: BMJ Open. 2025 Mar 3;15(3):e091929. doi: 10.1136/bmjopen-2024-091929 (PMC12010349; doi:10.1136/bmjopen-2024-091929)
Supplement: online supplemental file 4 [file bmjopen-15-3-s004.docx]

**Supplementary File 4:** Public involvement and dissemination floor based journey map
