# Supplementary material for: London’s Ultra Low Emission Zone and active travel to school: a qualitative study exploring the experiences of children, families and teachers
Source: BMJ Open. 2025 Mar 3;15(3):e091929. doi: 10.1136/bmjopen-2024-091929 (PMC12010349; doi:10.1136/bmjopen-2024-091929)
Supplement: Supplementary file 5 [file bmjopen-2024-091929.reviewer_comments.pdf]

PEER REVIEW HISTORY

BMJ Open publishes all reviews undertaken for accepted manuscripts. Reviewers are asked to complete a checklist review form and are provided with free text boxes to elaborate on their assessment. These free text comments are reproduced below.

ARTICLE DETAILS

Title (Provisional)

London’s Ultra Low Emission Zone and active travel to school: a qualitative study exploring the experiences of children, families and teachers

Authors

Alliott, Olivia; van Sluijs, Esther; Dove, Rosamund; Kalsi, Harpal; Mitchell, Jessica; Mudway, Ian; Randhawa, Gurch; Sartori, Luke; scales, james; Wood, Helen; Griffiths, Chris; Guell, Cornelia; Panter, Jenna

---

VERSION 1 - REVIEW

Reviewer 1

Name Nazar, Wojciech

Affiliation Faculty of Medicine, Medical University of Gdańsk, Marii Skłodowskiej-Curie 3a, 80-210 Gdańsk, Poland, Medical Faculty

Date 16-Aug-2024

COI I have no competing interests to declare

I would like to congratulate authors writing such an interesting and comprehensive paper.

The introduction describes the background of the study. The methods of this qualitative study are described thoroughly. The direct quotes from the interviews are included in the discussion and discussed correctly. The conclusions are written as a "one-liner" message, which is clear for the reader. The figures are detailed and clear.

The Standards for Reporting Qualitative Research (SRQR) Checklist is also filled out. Moreover, the authors include all other supporting files (Interview topic guides and vignettes) in the supplementary materials.

Overall, in my opinion the manuscript is well-written and does not require any changes prior to its publication.

Reviewer 2

Name okoye, ogochukwu

**Affiliation**	**Delta State University, Medicine**
**Date**	**30-Aug-2024**
**COI**	**None**

The research topic is relevant and apt. The authors have clearly presented a well-balanced account of the participants' opinions and the interpretations against the broader literature.

Find some comments below;

1. Page 9, Line 54: The authors noted that teachers were purposively selected based on their knowledge of school-based travel behaviour and policies; how was this determined?

2. Page 10, Line 27: What informed the sample size for this study (number of families and teachers selected)?

3. Page 11, Line 7: In whom was the pilot conducted? provide a brief description.

4. Page 11, Line 8: How was transcription done, any electronic software used or manually?

5. There was no record of debriefing the participants after data collection to ensure agreement with how authors' have presented/summarised their views.

6. Was any difference noted between views of online participants and face-face participants?

7. The discussion and conclusion were well written. However, from a realist's standpoint, it is important to highlight that regardless of the potential positive impacts of LEZ, some person will simply prefer to drive. For instance, some participants who may be socioeconomic advantaged (or not) have compliant cars and prefer/enjoy driving but the traffic mobility constraints is a source of displeasure. Policies should be inclusive and aim to satisfy vulnerable and non-vulnerable alike since all have equal rights.

8. lastly, there are some minor typos:

Page 6- Line 51: 'how' should replace 'now'

Page 6, Line 49: been suggested 'that' (missing word)

Page 11, Line 11 : which 'they' could take home.... (missing word)

Page 24, Line 22: we further 'illustrate' not 'illustration'

Page 33. Figure 2 is wrongly labelled as Figure 1

**Reviewer**	**3**
**Name**	**Gianquintieri, Lorenzo**
**Affiliation**	**Politecnico di Milano**
**Date**	**09-Sep-2024**

COI **I declare no competing interest.**

The proposed manuscript "London's Ultra Low Emission Zone and active travel to school: a qualitative study exploring the experiences of children, families and teachers" presents the results of an interview program aimed at assessing how the traffic limitations in the London area impacted the habits of families in relation to children's travel to school. The qualitative nature of the study, the very limited sample size (moreover selected with purposeful sampling), and the way results are presented, put the study in a more psycho-social research perspective, but it is not in line with medical/clinical requirements. In particular, main concerns are:

MAJOR

1) ABSTRACT: authors present contrasting results across different groups, depending on background, but it is not clear what condition leads to either of the identified outcomes (narrowing or exacerbation of inequities). A brief mention of such relationship would be beneficial.

2) INTRODUCTION: a thorough review of current literature, also considering analyses conducted in other cities with similar initiatives, is missing.

3) METHODS lines 54-58: "Teachers were recruited based on their knowledge of school-based travel behaviour and policies and interventions implemented in and around their schools" such purposeful selection may introduce a bias, as well-informed people might be oversensitive to positive effects. Was this accounted for (e.g. with general control group)?

4) "Families and teachers received a £20 voucher to thank them for their time" I think it is necessary that authors clarify why this was allowed by the ethical committee, since paying people enrolled in research appears not in line with ethical principles.

5) METHODS chapter 2.5 – Analysis: despite the standard structure followed and the criteria discussed in supplementary 3, the whole procedure appears too widely arbitrary to comply with medical research. Additionally, the description's clarity would benefit from a graphical scheme.

6) RESULTS: relevant concepts are reported in terms of quotations from single subjects, hence their statistical significance and robustness is not assessed. What makes them clinically relevant?

7) RESULTS: an overall resuming view of most relevant results would be desirable.

8) DISCUSSION: Many of the findings could be due to the confounding effect of the increased perceived safety generated by traffic reduction (as also presented in the results) rather than by the reduced pollution. This should be at least acknowledged in the limitations.

MINOR

9) There might be a typo in line 51 of introduction: “and now people responded”, I guess it is “how people responded”.

10) Methods line 45: please check the unclear sentence “The study compares children attending primary schools within or catchment areas within the central ULEZ area”.

11) Both figures are referred to in the caption as ‘figure 1’.

VERSION 1 - AUTHOR RESPONSE

Reviewer 1

I would like to congratulate authors writing such an interesting and comprehensive paper.

The introduction describes the background of the study. The methods of this qualitative study are described thoroughly. The direct quotes from the interviews are included in the discussion and discussed correctly. The conclusions are written as a "one-liner" message, which is clear for the reader. The figures are detailed and clear.
The Standards for Reporting Qualitative Research (SRQR) Checklist is also filled out.
Moreover, the authors include all other supporting files (Interview topic guides and vignettes) in the supplementary materials.
Overall, in my opinion the manuscript is well-written and does not require any changes prior to its publication.

Response: Thank you for your comments.

Reviewer 2

The research topic is relevant and apt. The authors have clearly presented a well-balanced account of the participants' opinions and the interpretations against the broader literature.

Find some comments below;

1. Page 9, Line 54: The authors noted that teachers were purposively selected based on their knowledge of school-based travel behaviour and policies; how was this determined?
Response: Thank you for this comment. We asked school staff to direct us to the most relevant and knowledgeable person in each school, as this varied between schools. Sometimes this was the headteacher, in other schools it was a teacher with responsibility for travel policy. We have added text to the manuscript on page 8 to clarify this.

2. Page 10, Line 27: What informed the sample size for this study (number of families and teachers selected)?
Response: We based on sample size on expectations for qualitative research which balances the need to be sufficiently large to sufficiently describe the phenomenon of interest and

address the research question, but not too large to risk repetitive data. The goal is to attain saturation.

3. Page 11, Line 7: In whom was the pilot conducted? provide a brief description.
Response: Thank you. We completed the pilot with one parent and one child and have added these details to page 9.

4. Page 11, Line 8: How was transcription done, any electronic software used or manually?
Response: Thank you. We have clarified that these interviews were transcribed manually by a third party on page 10.

5. There was no record of debriefing the participants after data collection to ensure agreement with how authors' have presented/summarised their views.
Response: Yes, that it is correct, we did not validate our interpretations with participants. We have added text to page 10 to clarify this.

6. Was any difference noted between views of online participants and face-face participants?
Response: This is interesting to raise, but there were no differences between face to face and online participants and these were a mix of parents and children and teachers who completed both types of interviews. We have added text to make this clear on page 9.

7. The discussion and conclusion were well written. However, from a realist's standpoint, it is important to highlight that regardless of the potential positive impacts of LEZ, some person will simply prefer to drive. For instance, some participants who may be socioeconomic advantaged (or not) have compliant cars and prefer/enjoy driving but the traffic mobility constraints is a source of displeasure. Policies should be inclusive and aim to satisfy vulnerable and non-vulnerable alike since all have equal rights.
Response: Thank you. We agree that our data collection methods might not have captured all possible explanations for effects (positive or negative). We acknowledge that there may be other explanations that were not voiced in our interviews in our strengths and limitations section on page 24.

8. lastly, there are some minor typos:
Page 6- Line 51: 'how' should replace 'now'
Page 6, Line 49: been suggested 'that' (missing word)
Page 11, Line 11 : which 'they' could take home.... (missing word)
Page 24, Line 22: we further 'illustrate' not 'illustration'
Page 33. Figure 2 is wrongly labelled as Figure 1
Response: Thank you for spotting. We have corrected these errors.

Reviewer: 3
Dr. Lorenzo Gianquintieri, Politecnico di Milano
Comments to the Author:
The proposed manuscript “London’s Ultra Low Emission Zone and active travel to school: a qualitative study exploring the experiences of children, families and teachers” presents the results of an interview program aimed at assessing how the traffic limitations in the London area impacted the habits of families in relation to children’s travel to school. The qualitative nature of the study, the very limited sample size (moreover selected with purposeful sampling), and the way results are presented, put the study in a more psycho-social research perspective, but it is not in line with medical/clinical requirements. In particular, main concerns are:

MAJOR

1) ABSTRACT: authors present contrasting results across different groups, depending on background, but it is not clear what condition leads to either of the identified outcomes (narrowing or exacerbation of inequities). A brief mention of such relationship would be beneficial.
Response: Thank you for the comment. In our abstract we highlight how the mechanisms that lead to these different outcomes and results differ by different contextual factors. We do not have the space in to the abstract to show how the different combinations of contextual factors led to different outcomes. We provide a high-level summary in the abstract. We also make the point that the ULEZ could exacerbate inequalities because the impacts differ by people’s existing travel mode and socioeconomic position.

2) INTRODUCTION: a thorough review of current literature, also considering analyses conducted in other cities with similar initiatives, is missing.
Thank you for the comment. Our introduction describes the current literature around the effectiveness of interventions to promote active travel to school, including several systematic reviews (page 2). The most recent review identifies no studies of low emission zones on children’s travel patterns and concludes that qualitative methods would be useful to elucidate how interventions may or may not work.

3) METHODS lines 54-58: “Teachers were recruited based on their knowledge of school-based travel behaviour and policies and interventions implemented in and around their schools” such purposeful selection may introduce a bias, as well-informed people might be oversensitive to positive effects. Was this accounted for (e.g. with general control group)?
Response: This purposeful sampling is normal in qualitative research.

4) “Families and teachers received a £20 voucher to thank them for their time” I think it is necessary that authors clarify why this was allowed by the ethical committee, since paying people enrolled in research appears not in line with ethical principles.
Response: This purposeful sampling is normal in qualitative research. We submitted our study plans and protocol to our ethics committee and this was approved. Providing incentives for research participants is known to increase participation and monetary incentives are ethically acceptable, as they may be viewed as a gesture of appreciation for the patient’s contributions, time, and effort (see Abdelazeem et al., 2022; The effectiveness of incentives for research participation: A systematic review and meta-analysis of randomized controlled trials. PlosOne.)

5) METHODS chapter 2.5 – Analysis: despite the standard structure followed and the criteria discussed in supplementary 3, the whole procedure appears too widely arbitrary to comply with medical research. Additionally, the description’s clarity would benefit from a graphical scheme.
Response: This is not medical research and the approaches to analysis are in line with qualitative approaches as described by Braun and Clarke (2021).

6) RESULTS: relevant concepts are reported in terms of quotations from single subjects, hence their statistical significance and robustness is not assessed. What makes them clinically relevant?
Response: This is normal for qualitative research. We understand the reviewer may be coming from a quantitative background where assessment of significance and clinical relevance is normal but this is not the focus of qualitative research.

7) RESULTS: an overall resuming view of most relevant results would be desirable.
Response: We provide a high level summary of themes from our work at the start of the results section.
“Common CMO configurations were identified which speak to how the ULEZ impacted children’s travel behaviour and journey to school. These configurations reflect narratives across two overarching, but intertwined, themes which were developed during the data analysis: i) how you travelled before the ULEZ impacts the experience of the ULEZ, ii) your socioeconomic position impacts the experience of the ULEZ” (page 13).

We then provide a summary figure (Figure 2) showing our key CMO configurations and then give more details in the rest of the results section to evidence our summary themes.

8) DISCUSSION: Many of the findings could be due to the confounding effect of the increased perceived safety generated by traffic reduction (as also presented in the results) rather than

by the reduced pollution. This should be at least acknowledged in the limitations.
Response: This qualitative research does not seek to establish the cause of the reduced pollution. These confounding effects are not relevant for qualitative research. In our limitations section we have acknowledged that the views of our participants might not fully capture all variations in the school journey and the ULEZ, but this is consistent with a qualitative approach, as noted on page 24.

MINOR

9) There might be a typo in line 51 of introduction: “and now people responded”, I guess it is “how people responded”.

10) Methods line 45: please check the unclear sentence “The study compares children attending primary schools within or catchment areas within the central ULEZ area”.

11) Both figures are referred to in the caption as ‘figure 1’.

Response: Thank you for noting these errors, some of which Reviewer 2 also identified.
